# Nuclear magnetic resonance spectroscopy reveals biomarkers of stroke recovery in a mouse model of obesity-associated type 2 diabetes

**DOI:** 10.1042/BSR20240249

**Published:** 2024-07-05

**Authors:** João P.P. Vieira, Dimitra Karampatsi, Ellen Vercalsteren, Vladimer Darsalia, Cesare Patrone, Joao M.N. Duarte

**Affiliations:** 1 Diabetes and Brain Function Unit, Department of Experimental Medical Science, Faculty of Medicine, Lund University, 221 84 Lund, Sweden; 2 Wallenberg Centre for Molecular Medicine, Lund University, 221 84 Lund, Sweden; 3 NeuroCardioMetabol Group, Department of Clinical Science and Education, Södersjukhuset, Internal Medicine, Karolinska Institutet, 118 83 Stockholm, Sweden

**Keywords:** biomarkers, blood, metabolomics, NMR, tMCAO

## Abstract

Obesity and Type 2 diabetes (T2D) are known to exacerbate cerebral injury caused by stroke. Metabolomics can provide signatures of metabolic disease, and now we explored whether the analysis of plasma metabolites carries biomarkers of how obesity and T2D impact post-stroke recovery. Male mice were fed a high-fat diet (HFD) for 10 months leading to development of obesity with T2D or a standard diet (non-diabetic mice). Then, mice were subjected to either transient middle cerebral artery occlusion (tMCAO) or sham surgery and allowed to recover on standard diet for 2 months before serum samples were collected. Nuclear magnetic resonance (NMR) spectroscopy of serum samples was used to investigate metabolite signals and metabolic pathways that were associated with tMCAO recovery in either T2D or non-diabetic mice. Overall, after post-stroke recovery there were different serum metabolite profiles in T2D and non-diabetic mice. In non-diabetic mice, which show full neurological recovery after stroke, we observed a reduction of isovalerate, and an increase of kynurenate, uridine monophosphate, gluconate and *N*6-acetyllysine in tMCAO relative to sham mice. In contrast, in mice with T2D, which show impaired stroke recovery, there was a reduction of *N,N*-dimethylglycine, succinate and proline, and an increase of 2-oxocaproate in serum of tMCAO versus sham mice. Given the inability of T2D mice to recover from stroke, in contrast with non-diabetic mice, we propose that these specific metabolite changes following tMCAO might be used as biomarkers of neurophysiological recovery after stroke in T2D.

## Introduction

Stroke is a leading cause of mortality and disability worldwide that is critically driven by dietary and environmental factors, and therefore there’s a pressing need for prevention of its emergence and recurrence [1]. Obesity and Type 2 diabetes (T2D) are associated with increased risk of developing cardio-metabolic pathology, including hypertension, myocardial infarction and stroke [2]. Importantly, T2D established before stroke is also a strong predictor of poor stroke recovery (see [3], and references there in), underlining the high need to identify new treatments to improve stroke recovery and reduce disability in people with T2D. In this perspective, in the present study, we aimed at exploring serum metabolite signatures induced by prolonged high-fat diet (HFD)-feeding leading to obesity with T2D, which reflects potential effects of metabolic disease and T2D on post-stroke functional recovery.

Metabolomics affords the measurement of many metabolites as a proximal reporter of disease and/or therapeutics [4,5]. Hence, metabolomics tools to profile metabolites in biological fluids can provide insight into metabolite signatures characteristic of T2D/obesity [6] and stroke [7]. Previous biomarkers from metabolomics studies in stroke patients included oxidized glutathione, kynurenine, glycine, glutamate and glutamine, glycolate, intermediaries of one-carbon metabolism (e.g., methylation reactions), such as folic acid, cysteine, S-adenosylhomocysteine, formate, the glycolysis end-products pyruvate and lactate, as well as certain lipids [8–11]. Interestingly, many of these stroke-induced metabolite changes in blood are also observed in obesity and T2D (see [12], and references therein). In this exploratory study, we tested the hypothesis that there are unique metabolic pathways impacted by stroke after long-term HFD-induced T2D/obesity.

Nuclear magnetic resonance (NMR) spectroscopy has been used in metabolomics-based studies [13]. Despite lower sensitivity than other analytical methods, namely mass spectrometry, ^1^H NMR spectroscopy offers the advantage of relatively simple sample processing, non-destructive character and high reproducibility, and predictable proportionality between signal and the concentration of nuclei within molecules of the sample [13]. Besides the quantitative analysis of metabolites, NMR spectra can be directly used as a fingerprint of metabolite concentrations that report the systemic metabolic status [12]. In the present study, a NMR spectroscopy-based metabolic fingerprinting approach was used to acquire metabolite profiles in serum. Multivariate statistical analysis was then employed to identify key metabolite concentration changes that might reflect the effect of T2D on stroke recovery.

## Material and methods

### Animals

Experiments were performed, according to the EU Directive 2010/63/EU, have been approved by the regional ethics committee (approval ID1126, Karolinska Institutet) and reported according to the ARRIVE guidelines [14]. The samples analyzed in the present study were obtained from a cohort of mice used in a previously published study [15]. Thus, no power calculations were performed for the present study. Briefly, male C57BL/6JRj mice (Janvier Labs, France) were housed in environmentally controlled conditions (22 ± 0.5°C, 12-h light/12-h dark cycle with access to food and water *ad libitum*). The mice were housed under pathogen free conditions in type III size individually ventilated cages with wood chip bedding, nest material and a cardboard tunnel. From 4 weeks of age, non-diabetic (ND) mice were fed a standard diet (SD) for 10 months, while mice in the T2D group were fed a high-fat diet (HFD) containing 60% energy from saturated fat. Obesity and T2D were confirmed by a body weight increase >20%, fasting glucose >7 mmol/L, hyperinsulinemia, and decreased insulin sensitivity (Supplementary Figure S1). Afterwards, mice were subjected to either transient (30 min) middle cerebral artery occlusion (tMCAO) or sham surgery under isoflurane anaesthesia, as detailed previously [15]. After tMCAO or sham surgery, all experimental groups were fed with SD during the 2 months of the recovery phase, to mimic the clinical setting of a balanced poststroke diet. Sample sizes in the current study were as follows (experimental unit): ND-sham (*n*=5), T2D-sham (*n*=4), ND-stroke (*n*=9) and T2D-stroke (*n*=9).

The present study analyzed serum samples obtained from terminal blood that were collected via cardiac puncture, after mice were anesthetized with an overdose of sodium pentobarbital [15]. Briefly, blood was left to clot for 20 min at room temperature, and then centrifuged for 15 min at 2000 × ***g***. Serum was collected and stored at −80°C.

### NMR spectroscopy

Serum metabolites were extracted as previously [16]. Briefly, serum (70 µ-l) and methanol were mixed at 1:3, vortexed and sonicated on ice for 30 min. After centrifugation at 13,000 × ***g*** and 4ºC for 30 min, supernatants were dried in a Savant SpeedVac concentrator (Thermofisher Scientific, Göteborg, Sweden) operating a room temperature. Dried samples were dissolved in 600 μl of D_2_O in 100 mmol/L sodium phosphate buffer, pH 7.4, with 0.01%(w/v) NaN_3_. Sodium fumarate (0.3 µmol) was added as internal standard, and samples were transferred into 5-mm Wilmad NMR tubes (Sigma-Aldrich, Germany). ^1^H NMR spectra were acquired on a Bruker Avance III HD 14.1 T spectrometer equipped with a standard TCI cryoprobe using the ZGPR pre-saturation pulse sequence for water suppression with spectral width of 9 kHz, 3 s acquisition time, relaxation delay of 22 s, and 256 acquisitions.

### Data analysis

NMR spectra were processed in MATLAB 2021b (MathWorks, Natick, MA U.S.A.) using the General NMR Analysis Toolbox (GNAT) [17]. After alignment of the fumarate peak to 6.5 ppm and phase adjustment, the real part of the spectra (spectral points between 0 and 12 ppm) were saved for analysis. After aligning spectra using fumarate as reference, we removed spectral points corresponding to chemical shifts of residual water (4.70–5.10 ppm), methanol (3.32–3.35 ppm), fumarate reference peak (6.45–6.58 ppm), and peaks of propylene glycol (anaesthetic vehicle; chemical shift ranges in ppm: 1.10–1.15, 3.40–4.43, 3.436–3.442, 3.509–3.521, 3.53–3.54, 3.841–3.884).

Retained data points were normalized to spectral area, and thresholded at 5-fold the noise level measured between 0 and 0.2 ppm. Any chemical shift with more than 50% of values below threshold was discarded. Peaks were identified by detection of local maxima, and these data points were used for subsequent analysis in SIMCA v.17.0.2 (Umetrics, Umeå, Sweden) by applying an automated orthogonal projection to latent structures (OPLS) regression on *z*-scores. OPLS regression models were validated by permutation tests (100 permutations for each analysis). OPLS model quality was assessed by the cumulative *R*^2^ (fraction of Y variation modelled) and cumulative *Q*^2^ (fraction of Y variation predicted by the model) obtained from SIMCA. Variable importance in projection (VIP) scores greater than 1 were considered important for the projection of the OPLS regression model.

**Figure 1:**
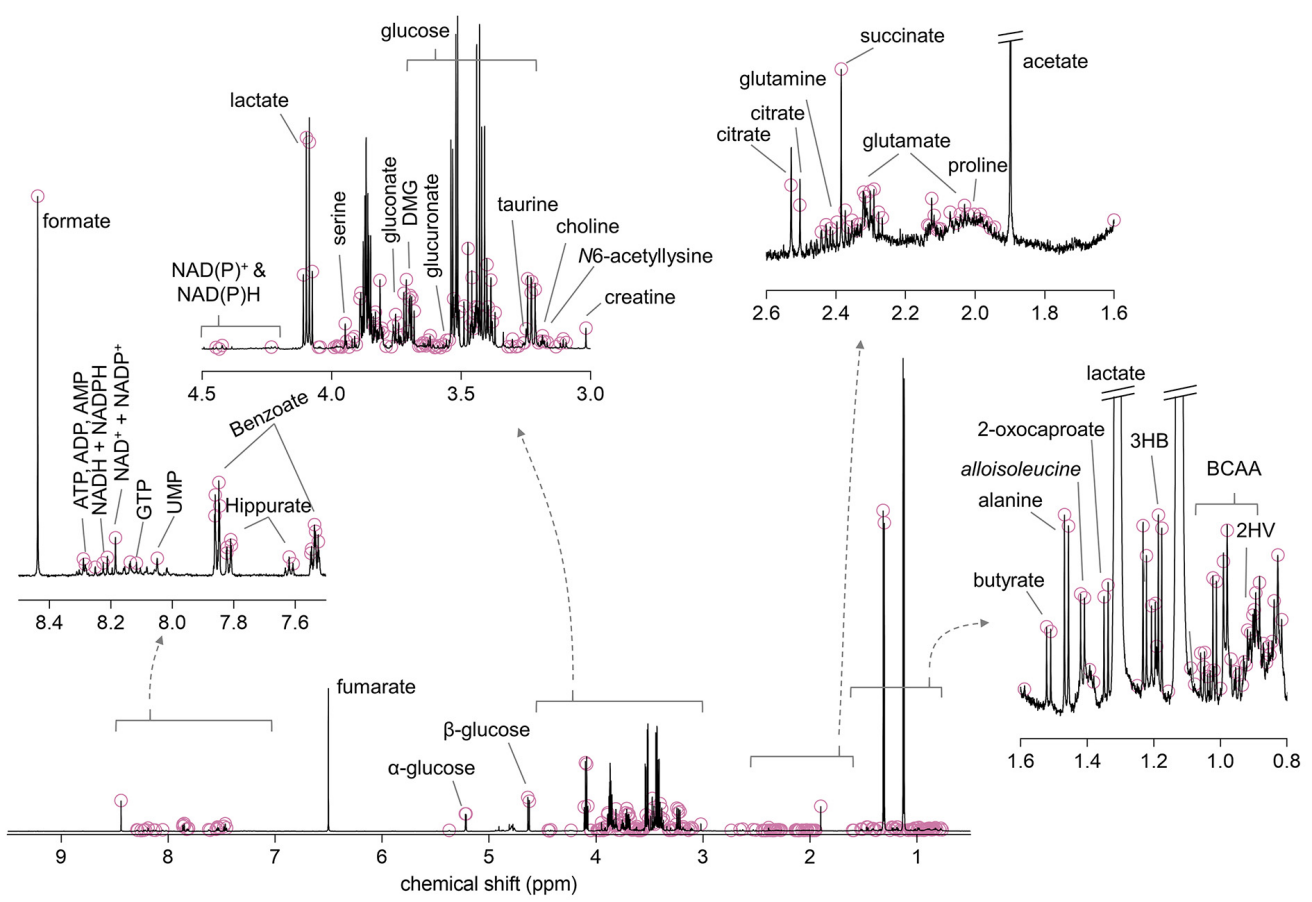
Typical ^1^H NMR spectrum of mouse plasma measured at 600 MHz The solid line represents the measured spectrum, and the circles depict the respective data points taken for OPLS analysis. Abbreviations: 3HB, 3-hydroxybutyrate; 2HV, 2-hydroxyvalerate; BCAA, branch chained amino acids.

Peaks were assigned to metabolites with Chenomx NMR Suite 9.0 (Chenomx, Edmonton, Alberta, Canada) and previous publications [18–22] (Figure 1). Peak amplitudes were considered to reflect metabolite concentrations, and z-scores of metabolites with VIP > 1 were used for pathway analysis in MetaboAnalyst 5.0 (www.metaboanalyst.ca), using the Kyoto Encyclopedia of Genes and Genomes (KEGG) as pathway library [23]. When multiple NMR spectral points were available, that with highest VIP score was used for pathway analysis.

MetaboAnalyst computes a *P-*value for the difference of each metabolic pathway (a group of functional-associated metabolites) rather than each metabolite alone, and employs a pathway topology analysis to determine the pathway impact score, which represents the importance of a given pathway in relation to the global metabolic network. False-discovery rate (FDR) was used to adjust *P-*values for false positives in the pathway analysis. Both adjusted *P*<0.05 and impact score >0 were used for identifying pathways of interest.

Means were compared with Student’s *t*-tests in Prism 9.5.1 (GraphPad, San Diego, CA, U.S.A.). Statistical results are available as supplementary material.

## Results

As previously reported (see [15]), HFD exposure for 10 months in this mouse cohort resulted in the establishment of a T2D phenotype with obesity over 25% BW increase, fasting hyperglycemia (fasting glucose over 7 mmol/L) and insulin resistance, when compared with SD feeding. After tMCAO neurological recovery was tracked by forepaw grip strength test and corridor test for 8 weeks. Results show that T2D significantly impaired post-stroke recovery [15].

### ND-stroke versus T2D-stroke mice

**Figure 2:**
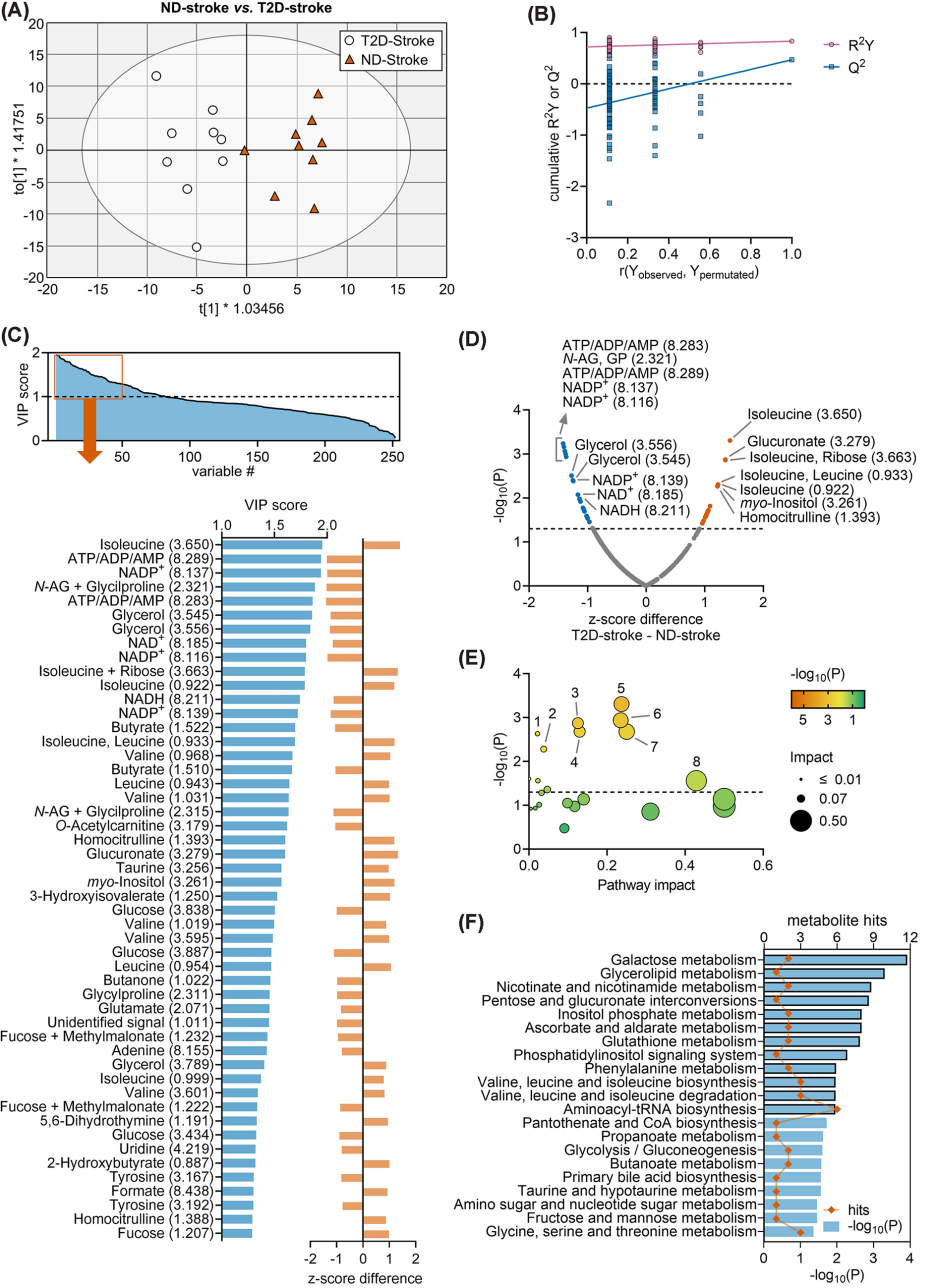
Multivariate data analysis of ND-stroke and T2D-stroke Multivariate data analysis using OPLS regression on NMR spectroscopy data obtained from serum samples of ND-stroke mice (*n*=9) or T2D-stroke mice (*n*=9). (**A**) Group separation in the OPLS score space. Ellipse represents Hotelling’s T2 (95%). (**B**) Response permutation test plot (*n*=100) for OPLS model validation. *R*^2^ and Q^2^ values corresponding to *y*-axis intercepts were 0.719 and −0.475, respectively. (**C**) VIP scores estimated from the OPLS model, and expansion of the top 50 chemical shifts with highest VIP score presented with the respective z-score difference for T2D-stroke - ND-stroke. (**D**) Volcano plot showing *z*-score differences and respective unadjusted  *P*-values resulting from *t*-tests. (**E,F**) Metabolic pathway analysis with MetaboAnalyst and the KEGG database on *z*-scores from metabolites with VIP > 1 in OPLS regression. Pathways with impact score = 0 are not represented in (E), but are listed in (F). Panels (**F**) include significant findings, that is, pathways with unadjusted *P*<0.05. Orange symbols and blue bars represent number of hits (metabolites analysed in that pathway) and −log_10_(*P*), respectively. Bars highlighted with black border indicate pathways which significance survives false discovery rate (FDR) correction. Dashed lines in panels A and C indicate  *P*=0.05. In C,D, labels are metabolite and chemical shift in ppm. Legend in panel (E): 1, Glutathione metabolism; 2, Phosphatidylinositol signaling system; 3, Pentose and glucuronate interconversions; 4, Inositol phosphate metabolism; 5, Glycerolipid metabolism; 6, Nicotinate and nicotinamide metabolism; 7, Ascorbate and aldarate metabolism; 8, Taurine and hypotaurine metabolism. Abbreviations: n-AG, N-Acetylglutamine; GP, glycylproline.

Since ND mice recover from stroke while T2D mice show limited neurophysiological recovery [15], we set to identify differences in metabolite concentrations that are likely associated with impaired stroke recovery. Thus, we first explored whether T2D mice have a distinct metabolite profile after recovery from stroke, when compared with ND mice. The OPLS regression applied to NMR data generated a model composed of 1 predictive component and 1 orthogonal component with cumulative *R*^2^ = 0.831 and *Q*^2^ = 0.469, and allowed good group separation in the OPLS component space (Figure 2A). OPLS model validation resulted in *R*^2^ = 0.719 and *Q*^2^ = −0.475 (Figure 2B). A total of 81 chemical shifts had VIP > 1 (Figure 2C), with the highest VIP scores corresponding to, e.g., glycerol, adenine nucleotides, NAD^+^/NADP^+^, leucine, isoleucine, valine, butyrate, *O*-acetylcarnitine, homocitrulline, glucoronate, taurine, *myo*-inositol, or 3-hydroxyisovalerate. Multiple *t*-tests comparing average *z*-scores in HFD-stroke and ND-stroke mice confirmed these metabolite alterations (Figure 2D).

Pathway analysis revealed several metabolic pathways that were impacted by T2D in mice that were subjected to stroke (Figure 2E,F). Altered pathways (adjusted *P*<0.05; impact score>0) in T2D versus ND mice after stroke recovery included glutathione metabolism, pentose and glucuronate interconversions, inositol phosphate metabolism and phosphatidylinositol signaling, glycerolipid metabolism, nicotinate and nicotinamide metabolism, ascorbate and aldarate metabolism.

### ND-stroke versus ND-sham mice

**Figure 3:**
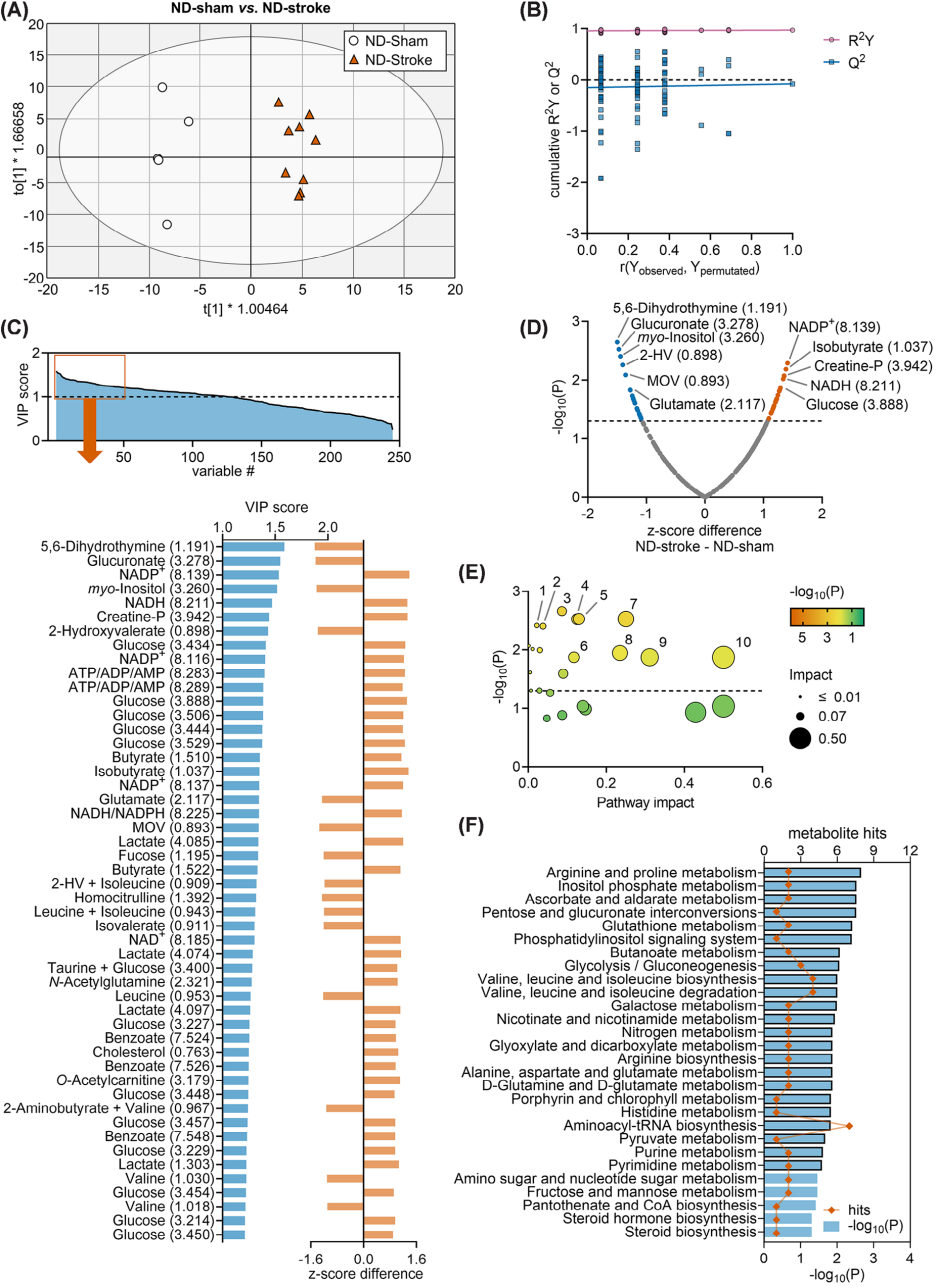
Multivariate data analysis of ND-sham and ND-stroke Multivariate data analysis using OPLS regression on NMR spectroscopy data obtained from serum samples of SD-fed mice subjected to stroke (*n*=9) or sham (*n*=5) surgery. (**A**) Group separation in the OPLS score space. Ellipse represents Hotelling’s T2 (95%). (**B**) Response permutation test plot (*n*=100) for OPLS model validation. *R*^2^ and *Q*^2^ values corresponding to *y*-axis intercepts were 0.956 and −0.149, respectively. (**C**) VIP scores estimated from the OPLS model, and expansion of the top 50 chemical shifts with highest VIP score presented with the respective z-score difference for ND-stroke - ND-sham. (**D**) Volcano plot showing *z*-score differences and respective unadjusted *P*-values resulting from *t*-tests. (**E,F**) Metabolic pathway analysis with MetaboAnalyst and the KEGG database on z-scores from metabolites with VIP > 1 in OPLS regression. Pathways with impact score = 0 are not represented in (E), but are listed in (F). Panel (**F**) includes all significant findings, that is, pathways with unadjusted *P*<0.05. Orange symbols and blue bars represent number of hits (metabolites analysed in that pathway) and −log_10_(*P* ), respectively. Bars highlighted with black border indicate pathways which significance survives FDR correction. Dashed lines in panels D and E indicate *P*=0.05. In C,D, labels are metabolite and chemical shift in ppm. Legend in panel (**E**): 1, Glutathione metabolism; 2, Phosphatidylinositol signaling system; 3, Arginine and proline metabolism; 4, Pentose and glucuronate interconversions; 5, Inositol phosphate metabolism; 6, Arginine biosynthesis; 7, Ascorbate and aldarate metabolism; 8, Nicotinate and nicotinamide metabolism; 9, Alanine, aspartate and glutamate metabolism; 10, D-Glutamine and D-glutamate metabolism. Abbreviations: 2-HV, 2-Hydroxyvalerate; Glu, glutamate; MOV, 3-methyl-2-oxovalerate.

In order to identify metabolite changes associated with normal stroke recovery, we then tested the effects of stroke in ND mice, that is, in the absence of obesity-induced T2D. The OPLS regression applied to NMR data generated a model composed of 1 predictive component and 3 orthogonal components with cumulative *R*^2^ = 0.969 and *Q*^2^ = −0.0777, and allowed good group separation in the OPLS component space (Figure 3A). OPLS model validation by permutation analysis resulted in *R*^2^ = 0.956 and *Q*^2^ = −0.149 (Figure 3B). This relatively high *Q*^2^ indicated model overfitting in OPLS, and while it allows identifying metabolites of importance in discriminating the experimental groups, it should not be used in a predictive manner. A total of 125 chemical shifts had VIP > 1 (Figure 3C), with the highest VIP scores corresponding to 5,6-dihydrothymine, glucoronate, NAD^+^/NADP^+^, *myo*-inositol, creatine phosphate, 2-hydroxyvalerate, glucose, adenine nucleotides (ATP/ADP/AMP), butyrate, isobutyrate, 3-methyl-2-oxovalerate, lactate or fucose. Multiple *t*-tests comparing average *z*-scores in ND-sham and ND-stroke mice confirmed several of these metabolite alterations (Figure 3D).

Pathway analysis revealed several metabolic pathways that were impacted by stroke, that is, were significantly different between ND-sham and ND-stroke mice (Figure 3E,F). Altered pathways after post-stroke recovery included glutathione metabolism, inositol phosphate metabolism and phosphatidylinositol signaling, arginine biosynthesis/arginine and proline metabolism, pentose and glucuronate interconversions, ascorbate and aldarate metabolism, nicotinate and nicotinamide metabolism, metabolism of amino acids (alanine, aspartate, glutamine, glutamate, valine, leucine and isoleucine), galactose metabolism, glycolysis/gluconeogenesis, and metabolism of pyrimidines and purines.

### T2D-stroke versus T2D-sham mice

**Figure 4:**
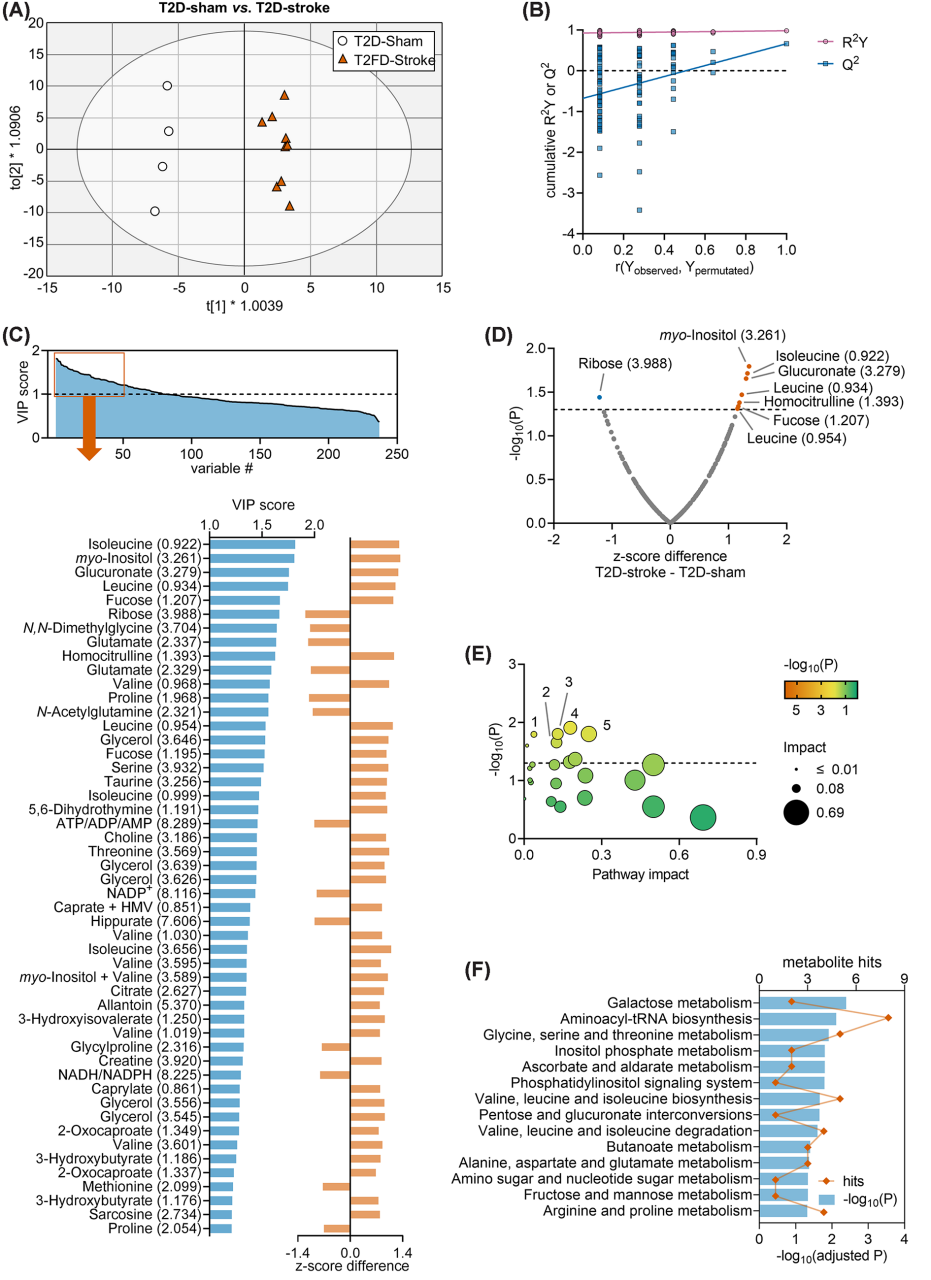
Multivariate data analysis of T2D-sham and T2D-stroke Multivariate data analysis using OPLS regression on NMR spectroscopy data obtained from serum samples of T2D-stroke (*n*=9) or T2D-sham (*n*=4). (**A**) Group separation in the OPLS score space. Ellipse represents Hotelling’s T2 (95%). (**B**) Response permutation test plot (*n*=100) for OPLS model validation. *R*^2^ and *Q*^2^ values corresponding to *y*-axis intercepts were 0.926 and −0.678, respectively. (**C**) VIP scores estimated from the OPLS model, and expansion of the top 50 chemical shifts with highest VIP score presented with the respective *z*-score difference for T2D-stroke - T2D-sham. (**D**) Volcano plot showing *z*-score differences and respective unadjusted *P*-values resulting from *t*-tests. (**E,F**) Metabolic pathway analysis with MetaboAnalyst and the KEGG database on *z*-scores from metabolites with VIP > 1 in OPLS regression. The plot depicts pathway impact score and respective significance (uncorrected *P*-values). Results with pathway impact of 0 are not represented in (E) but are listed in (**F**). Dashed lines in panels D and E indicate *P*=0.05. In C,D, labels are metabolite and chemical shift in ppm. Abbreviations: HMV, 2-Hydroxy-3-methylvalerate. Legend in (E): 1, Phosphatidylinositol signaling system; 2, Pentose and glucuronate interconversions; 3, Inositol phosphate metabolism; 4, Glycine, serine and threonine metabolism; 5, Ascorbate and aldarate metabolism.

In addition to metabolite differences between ND-stroke and T2D-stroke mice (see Figure 2), we set to explore metabolite concentration changes that are activated by stroke followed by impaired recovery in T2D. Thus, for identifying metabolite changes associated with impaired post-stroke recovery, we compared T2D mice subjected to stroke versus sham (both fed HFD prior to stroke). The OPLS regression applied to NMR data generated a model composed of 1 predictive component and 3 orthogonal components with cumulative *R*^2^ = 0.981 and *Q*^2^ = 0.663, and allowed good group separation in the OPLS component space (Figure 4A). OPLS model validation by permutation analysis resulted in *R*^2^ = 0.926 and *Q*^2^ = −0.678 (Figure 4B), indicating that it is significantly different from a random model, and likely suitable to identifying biomarkers. A total of 83 chemical shifts showed VIP > 1 (Figure 4C), with the highest VIP scores corresponding to isoleucine, *myo*-inositol, glucoronate, leucine, fucose, ribose, *N,N*-dimethylglycine, glutamate, homocitrulline, valine, proline, *N*-acetylglutamine, glycerol, serine, and taurine. Multiple *t*-tests comparing average *z*-scores in T2D-sham and T2D-stroke mice confirmed several of these metabolite alterations (Figure 4D).

Group average *z*-scores from metabolites with VIP > 1 in the OPLS regression were then used for pathway analysis in MetaboAnalyst. Between T2D-sham and T2D-stroke mice, no metabolic pathways survived false-discovery rate (FDR) correction at *P*<0.05, but one can observe a tendency for an impact on inositol phosphate metabolism and phosphatidylinositol signaling, pentose and glucuronate interconversions, glycine, serine and threonine metabolism, and ascorbate and aldarate metabolism (Figure 4E,F).

### ND-sham versus T2D-sham mice

Aiming at pinpointing metabolite changes that were specifically linked to stroke recovery, we finally set to test which metabolites and pathway changes mainly reflect effects of obesity/T2D. Such metabolic changes associated with T2D are likely to occur already before stroke, and not exclusively associated with recovery. However, they possibly play a detrimental role in impaired recovery after stroke.

**Figure 5:**
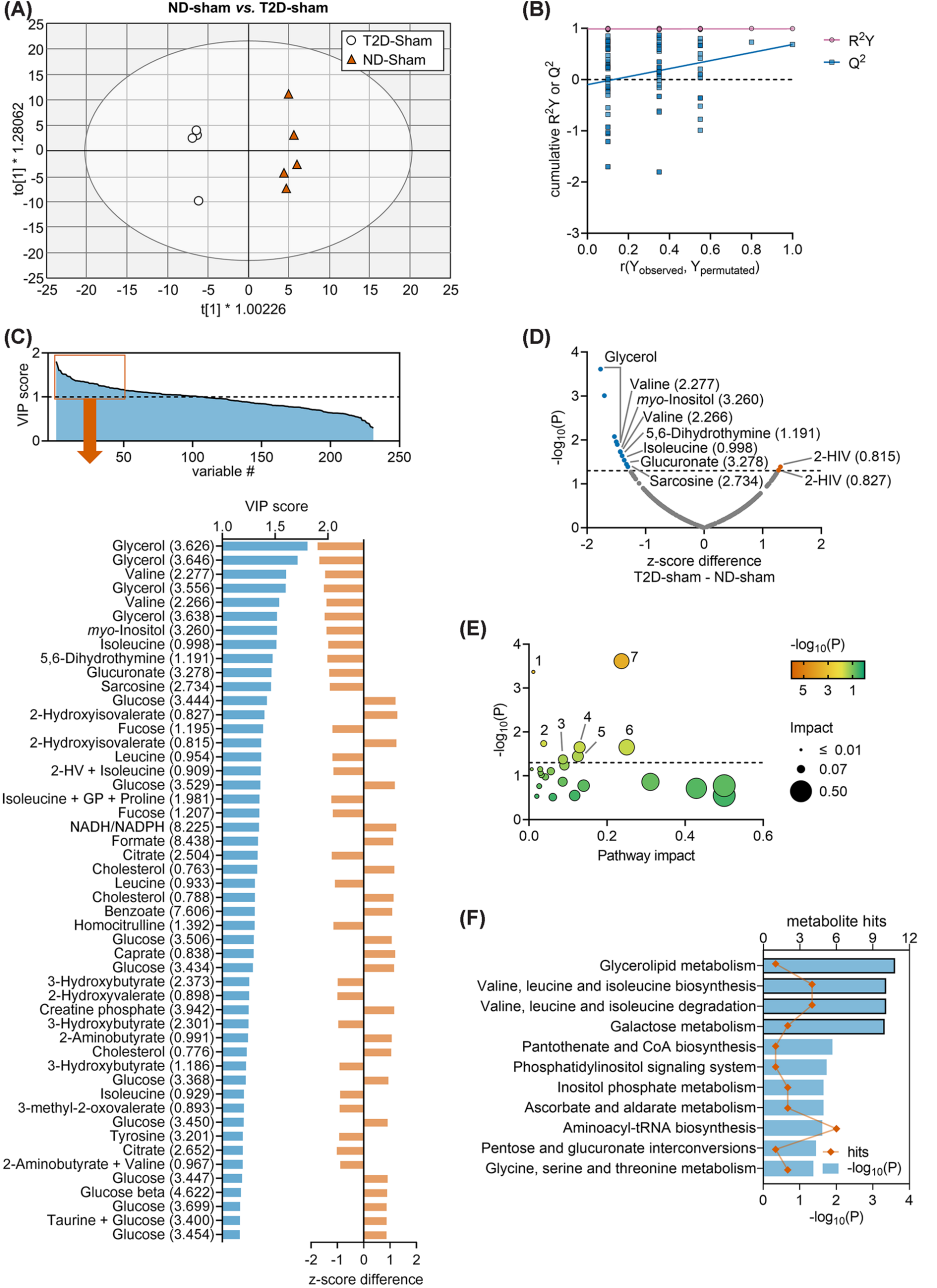
Multivariate data analysis of ND-sham and T2D-sham Multivariate data analysis using OPLS regression on NMR spectroscopy data obtained from serum samples of ND-sham mice (*n*=5) or T2D-sham mice (*n*=4). (**A**) Group separation in the OPLS score space. Ellipse represents Hotelling’s T2 (95%). (**B**) Response permutation test plot (*n*=100) for OPLS model validation. *R*^2^ and *Q*^2^ values corresponding to *y*-axis intercepts were 0.988 and −0.100, respectively. (**C**) VIP scores estimated from the OPLS model, and expansion of the top 50 chemical shifts with highest VIP score presented with the respective *z*-score difference for T2D-sham - ND-sham. (**D**) Volcano plot showing *z*-score differences and respective unadjusted *P*-values resulting from *t*-tests. (**E,F**) Metabolic pathway analysis with MetaboAnalyst and the KEGG database on *z*-scores from metabolites with VIP > 1 in OPLS regression. Pathways with impact score = 0 are not represented in (E), but are listed in (F). Panels (F) include significant findings, that is, pathways with unadjusted *P*<0.05. Orange symbols and blue bars represent number of hits (metabolites analysed in that pathway) and −log_10_(*P*), respectively. Bars highlighted with black border indicate pathways which significance survives false discovery rate (FDR) correction. Dashed lines in panels D and E indicate *P*=0.05. In C,D, labels are metabolite and chemical shift in ppm. Legend in panel (E): 1, Valine, leucine and isoleucine degradation; 2, Phosphatidylinositol signaling system; 3, Glycine, serine and threonine metabolism; 4, Inositol phosphate metabolism; 5, Pentose and glucuronate interconversions; 6, Ascorbate and aldarate metabolism; 7, Glycerolipid metabolism. Abbreviations: 5,6-DiHT, 5,6-Dihydrothymine; 2-HIV, 2-Hydroxyisovalerate; 2-HV, 2-Hydroxyvalerate; GP, glycylproline.

Thus, we compared T2D-sham versus ND-sham, that is, groups without ischemic brain damage. The OPLS regression applied to NMR data generated a model composed of 1 predictive component and 3 orthogonal components with cumulative *R*^2^ = 0.993 and *Q*^2^ = 0.683, and allowed good group separation in the OPLS component space (Figure 5A). The permutation analysis resulted in *R*^2^ = 0.988 and *Q*^2^ = −0.100 (Figure 5B). Once again, while explaining the observed data, this relatively high Q2 precludes predicting HFD effects by the obtained model. A total of 107 chemical shifts had VIP > 1 (Figure 5C), with the highest VIP scores corresponding to, e.g*.*, glycerol, valine, *myo*-inositol, leucine, isoleucine, 5,6-dihydrothymine, glucoronate, sarcosine, glucose, 2-hydroxyisovalerate, 2-hydroxyvalerate, fucose, NADH/NADPH, formate or citrate. Multiple *t*-tests comparing average *z*-scores in ND-sham and T2D-sham mice confirmed several of these metabolite alterations (Figure 5D).

Pathway analysis revealed that glycerolipid metabolism, and metabolism of valine, leucine and isoleucine were the main pathways impacted by T2D in sham mice (Figure 5E,F).

### Uniquely altered pathways after stroke

From the four analyses described above, we could observe metabolic changes induced by T2D, stroke or both. By understanding which pathways can be ascribed to T2D *per se*, we expected to highlight those linked to either normal or impaired stroke recovery.

**Figure 6:**
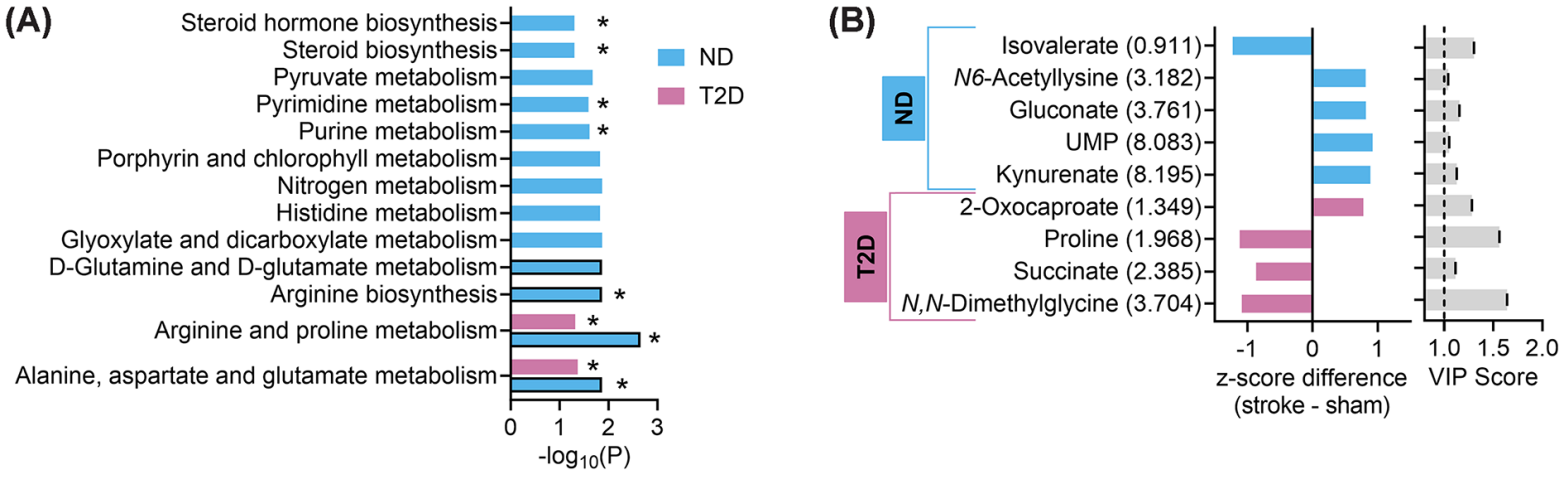
Uniquely altered pathways and metabolites Identification of uniquely altered pathways (**A**) and metabolites with VIP > 1 (**B**) in stroke versus sham mice. Panel A represents pathways that are significantly altered in stroke vs. sham comparisons but not in HFD vs. SD comparisons. Bars highlighted with black border indicate pathways which significance survives false discovery rate (FDR) correction (adjusted *P*<0.05). Pathways with impact score >0 are identified by *. Panel B shows unique metabolite changes in stroke vs. sham mice, and their VIP score in the respective comparison. Metabolites are shown with the respective chemical shifts (in ppm). Abbreviation: UMP, uridine monophosphate.

Some pathways were specifically modified by stroke and following recovery, and not associated with T2D (Figure 6A). To our surprise, although stroke resulted in several pathway alterations, none of these was specific to T2D-stroke. Instead, stroke-induced alterations in ND-stroke *versus* ND-sham were mostly absent when comparing T2D-stroke versus T2D-sham (Figure 6A). The KEGG pathways that remained altered in T2D-stroke versus T2D-sham mice were the metabolism of arginine and proline, and the metabolism of the amino acids alanine, aspartate, glutamate and glutamine.

We also explored which metabolite concentrations were uniquely impacted by stroke and not T2D/obesity (Figure 6B). In ND mice, unique concentration changes contributing to explaining stroke effects were a reduction of isovalerate, and an increase of kynurenate, UMP, gluconate and *N6*-acetyllysine. In turn, unique concentration changes explaining stroke effects in T2D mice were a reduction of *N,N*-dimethylglycine, succinate and proline, and an increase of 2-oxocaproate.

## Discussion

The present study explored potential alterations to the NMR spectral profile of serum metabolites in ND and T2D/obese mice subjected to tMCAO followed by 2 months of recovery. The experimental design included prolonged HFD exposure pre-tMCAO, which resulted in obesity (over 25% body-weight increase) as well as hyperglycemia and insulin resistance, the main features of T2D [15]. The present work confirmed metabolite signatures induced by HFD-feeding leading to T2D/obesity, and identified metabolite changes linked to the 2-month recovery phase following a stroke event.

During post-stroke recovery, there were much less metabolite alterations in the serum of T2D/obese mice than of ND mice. Moreover, impacted metabolic pathways in post-stroke recovery in T2D/obese mice (versus respective sham) failed to survive correction for multiple comparisons. Since post-stroke functional recovery was worse in T2D than ND mice [15], we suggest that systemic metabolic alterations observed non-T2D mice are crucial for improving stroke recovery. However, our study does not pinpoint causal relationships between metabolism and stroke recovery, and further research is warranted.

The most significant metabolic pathways uniquely associated with stroke recovery in ND mice were arginine and proline metabolism, and metabolism of the amino acids alanine, aspartate, glutamate and glutamine. While circulating amino acid levels are among the most important metabolite alterations after stroke, there’s limited research on their participation in post-stroke recovery. A recent study demonstrated significant differences in proline, arginine and glutamate levels in non-fasting venous blood between individuals with good and poor recovery after stroke [24]. Moreover, the levels of serum arginine were predictive of the recovery rate after stroke [24]. Arginine metabolism generates nitric oxide, and strategies to restore nitric oxide-generating pathways have been proposed as a mean of improving cerebral blood flow and functional outcomes after cerebral ischemia [25].

Increased glutamate release from depolarized ischemic neurons causes excitotoxicity and becomes an injury driver. Moreover, increased glutamate levels are a risk factor for cardiovascular disease and stroke in particular [26]. Thus, glutamate scavenging is proposed for improving post-stroke recovery [27]. Interestingly, independently of pre-stroke diet type, we observed lower levels of glutamate in mice recovering from tMCAO when compared with mice with sham surgery. Our results, however, refer to serum samples acquired at 2 months after the stroke event (at the end of the post-stroke recovery phase) and are not representative of the acute phase of stroke where augmented brain glutamate release could be expected.

Proline metabolism appears among the most importantly altered pathways in stroke recovery. More specifically, proline levels were found lower in individuals with poorer recovery after stroke [24]. Accordingly, in our study, mice recovering from tMCAO had less serum proline than sham mice, and proline was found to be an important metabolite (VIP > 1) only when comparing stroke *versus* sham in the T2D/obese mice, unlike the ND mice.

In sum, since T2D/obese mice do not fully recover after tMCAO [15], these altered pathways identified only in lean, ND mice are likely important for improved recovery after stroke.

We further explored specific metabolite alterations after tMCAO in either ND or T2D/obese mice, that is, metabolite alterations that do not overlap between the two comparisons. In ND-stroke mice versus ND-sham there was a reduction in levels of isovalerate, which is a short-chain fatty acid without apparent relation to stroke outcomes [28,29]. Some of our findings have already been suggested by previously published studies. Namely, levels of *N*6-acetyllysine were higher in ND-stroke than ND-sham mice, but stroke-induced changes on this metabolite were not identified in T2D/obesity. It has been reported that levels of metabolites of lysine metabolism such as *N*6-acetyllysine are reduced in both diabetic and non-diabetic individuals that are at high risk of stroke [30]. We also observed that the levels of kynurenic acid were higher in ND-stroke than ND-sham mice. The kynurenine pathway is known to be involved in stroke, with the kynurenic acid having neuroprotective properties [31].

While these alterations after tMCAO in non-T2D mice might be related to stroke recovery, it is plausible that metabolite alterations identified after tMCAO in T2D/obesity are linked to deleterious effects preventing stroke recovery. For example, we found lower levels of proline and succinate in the HFD-stroke group than the HFD-sham group. Accordingly, proline levels were reported lower in individuals with poorer recovery after stroke [24,32]. Succinate is known to accumulate in ischemia, and its subsequent metabolism upon reperfusion drives superoxide production in mitochondria. Therefore, targeting succinate metabolism has been proposed for reduction of brain injury [32].

In our study, the levels of *N,N*-dimethylglycine were also lower in the HFD-stroke group compared with HFD-sham. *N,N*-dimethylglycine is a product from choline metabolism that can act as an agonist of the glycine site of the NMDA receptor, exerting neuromodulatory effects [33]. Dimethylglycine is proposed to mediate positive effects of intermittent fasting on neurological disease in a model of Alzheimer’s disease [34]. Exogenous delivery of dimethylglycine has also been proposed to enhance the activity of cellular antioxidant systems and, thus, preventing oxidative stress [35]. On the other hand, low levels of dimethylglycine appeared linked to the development of metabolic disease [36]. Therefore, reduced dimethylglycine could contribute to poor recovery from stroke in HFD-fed mice.

## Limitations

Our results revealed several metabolites that were important in differentiating recovery from tMCAO from sham surgery, but no previous relation has been established between them and stroke. Noteworthy, many of these metabolites appear in serum at low concentrations and they represent small peaks in our NMR spectra or show complex metabolite signals that substantially overlap in the spectra. This is an important factor to be considered and was taken into account during peak assignment to metabolites. Moreover, overlapping peaks were not used for pathway analysis since one cannot determine which metabolite resulted in the observed signal change. In future studies, peak assignment could be improved through 2D NMR spectroscopy experiments. Targeted mass spectrometry approaches should also be considered for increasing sensitivity for detection of low concentrated metabolites in serum.

Another important limitation of our study is the small sample size in sham groups. However, this study was meant to be an exploration of potential spectral signatures and metabolites that could provide clues on metabolic pathways to be explored for understanding the impact of T2D/obesity and metabolic syndrome in the post-stroke recovery. In addition, studies of the identified metabolites should also focus on the acute phase of stroke, and test whether they predict future stroke recovery.

Finally, we must acknowledge that translational research from mice to humans in metabolomics studies is limited by species differences in metabolism and physiology, as well as by differential progression of recovery after stroke and response to treatments. These differences can lead to distinct metabolite profile trajectories after stroke and, therefore, the relevance of the serum biomarkers here identified needs to be further investigated in human samples. Nevertheless, the pathways associated with recovery after stroke are likely the same across species. Noteworthy, these pathways are not necessarily altered within the brain lesion, but could be associated with metabolic and inflammatory processes in peripheral tissues [37].

## Conclusion

Our study demonstrates the potential of NMR spectroscopy to detect signals of potential biomarkers associated with normal/physiological and impaired stroke recovery. Thus, signals in the NMR spectrum of blood fractions such as serum or plasma deserve further investigation for putative unbiased stratification and prediction of stroke recovery trajectories, namely in the context of T2D.

## Supplementary Material

Supplementary Figure S1 and Table

## Data Availability

The data that support the findings of this study are available from the corresponding author upon reasonable request.

## Open Access

Open access for this article was enabled by the participation of Lund University in an all-inclusive *Read & Publish* agreement with Portland Press and the Biochemical Society under a transformative agreement with Bibsam.

## Abbreviations

FDR: false discovery rate
HFD: high-fat diet
KEGG: Kyoto Encyclopedia of Genes and Genomes
LC: liquid chromatography
MS: mass spectrometry
ND: non-diabetic
NMR: nuclear magnetic resonance
OPLS: orthogonal projection to latent structures
SAH: *S*-adenosylhomocysteine
T2D: Type 2 diabetes mellitus
tMCAO: transient middle cerebral artery occlusion
UMP: uridine monophosphate
