# Supplementary material for: Nuclear magnetic resonance spectroscopy reveals biomarkers of stroke recovery in a mouse model of obesity-associated type 2 diabetes
Source: Biosci Rep. 2024 Jul 5;44(7):BSR20240249. doi: 10.1042/BSR20240249 (PMC11230867; doi:10.1042/BSR20240249)

Supplementary figure S1. Study design, metabolic phenotype, and neurological assessment. (A) T2D mice and ND mice were fed HFD and SD, respectively, during 10 months. After, stroke was induced by 30 minutes of tMCAO, and mice were fed standard diet for 2 months. (B) Body weight, (C) fasting blood glucose, (D) blood glucose during an insulin tolerance test (ITT), and (E) area under the curve (AUC) of the ITT in (D) before 30-minute tMCAO or sham surgery. (F) Grip strength measured after 3 days post-tMCAO and weekly until the end of the study, and (G) grip strength recovery measured as AUC of (F). (H) Body weight, (I) fasting glucose, and (J) area under the curve (AUC) of and ITT during standard diet feeding following tMCAO or sham surgery. Data is described as mean±SD. $^{**}P<0.01$, $^{****}P<0.0001$ in t-test comparisons, or in Šídák's multiple comparisons test following significant effects of T2D or significant T2D*time interaction in ANOVA. Data and experimental details were previously reported by Karampatsi *et al*. [15].

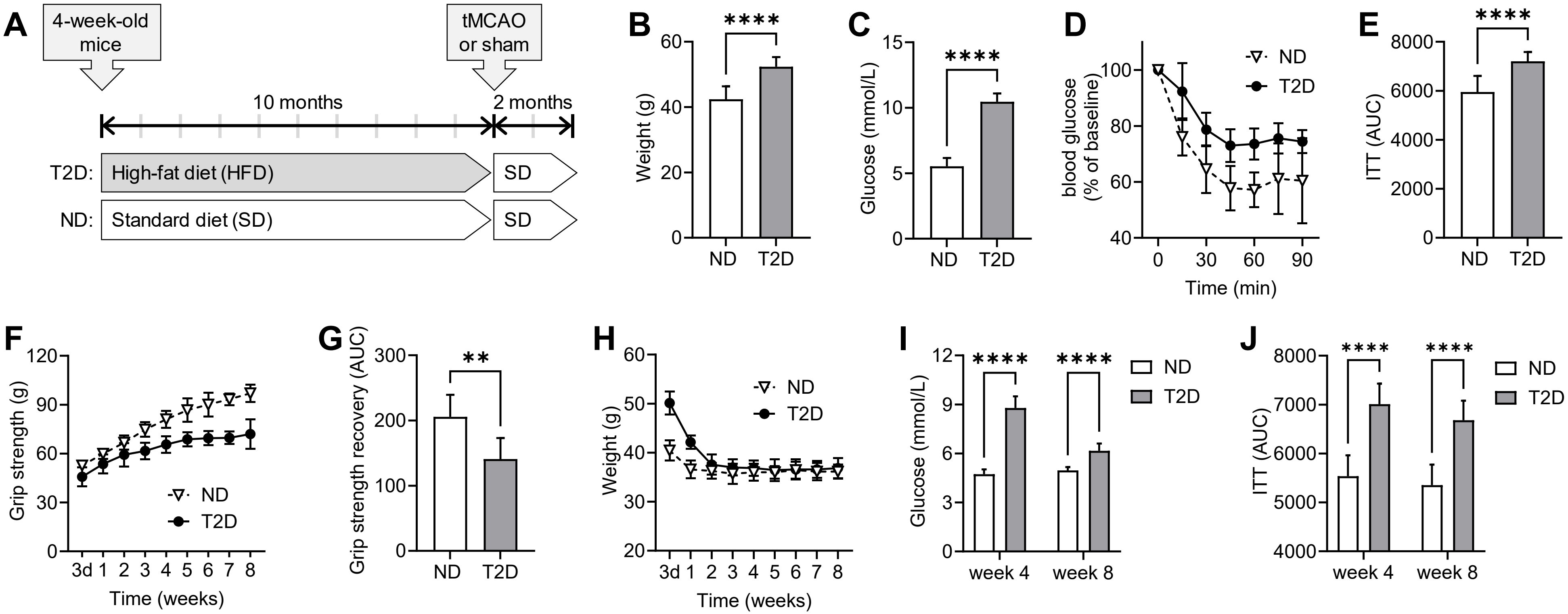

Supplement: Supplementary Figure S1 and Table [file BSR-2024-0249_supp.zip › BSR-2024-0249_supps1.pdf]
